# Whole-life body composition trajectory and longevity: role of insulin

**DOI:** 10.18632/aging.202727

**Published:** 2021-03-19

**Authors:** Yu-Hsuan Lin, Shiow-Chwen Tsai, Sheng-Ju Chuang, M. Brennan Harris, Kunanya Masodsai, Pei-Ni Chen, Chao-Chieh Hsieh, Theodore Killian, Chih-Yang Huang, Chia-Hua Kuo

**Affiliations:** 1 Laboratory of Exercise Biochemistry, University of Taipei, Taipei 111, Taiwan; 2 Department of Kinesiology and Health Science, College of William and Mary, Williamsburg, VA 23187, USA; 3 Cardiovascular and Mitochondrial Related Disease Research Center, Hualien Tzu Chi Hospital, Buddhist Tzu Chi Medical Foundation, Hualien 970, Taiwan; 4 Center of General Education, Buddhist Tzu Chi Medical Foundation, Tzu Chi University of Science and Technology, Hualien 970, Taiwan; 5 Graduate Institute of Biomedical Sciences, China Medical University, Taichung 404, Taiwan; 6 Department of Medical Research, China Medical University Hospital, China Medical University, Taichung 404, Taiwan; 7 Department of Medical Laboratory Science and Biotechnology, Asia University, Taichung 413, Taiwan; 8 De Duve Insitute, Université Catholique de Louvain (UCL), Woluwe-Saint-Lambert B-1200, Brussels, Belgium

**Keywords:** sarcopenia, osteopenia, longevity, frailty, cachexia

## Abstract

The present study assessed the body composition trajectory of rats (N = 96) placed into 5 groups according to lifespan, using dual-energy x-ray absorptiometry every 6 months until end-of-life. A striking linearity between lifespan and bone mass percentage (not absolute bone mass) was observed. Long-lived rats show a higher bone mass percentage with a delayed insulin rise to a similar peak level as short-lived counterparts, followed by insulin declines and bone mass loss. Decreasing insulin after streptozotocin (STZ) injection caused a rapid bone mass loss (-10.5%) with a decreased 5-day survival rate to 35% in old rats (20 months). Insulin replacement to STZ-injected rats completely blocked bone mass loss and increased the survival rate to 71%. Normal old rats (20 months) had faster lean mass loss despite greater myofiber regeneration (centronucleation) compared with the young rats (4 months). Increased CD68^+^ and CD163^+^ cell infiltration into insulin-depleted muscle suggests a bone marrow cell exhaustion by aging muscle. Bone produces stem cells and phagocytes to continuously rejuvenate peripheral tissues. Our data suggests that aging and unsustainable life is associated with development of disproportionality between bone and the growing body size, partly due to insulin reversal from hyperinsulinemia during late life.

## INTRODUCTION

Multicellular organisms may be viewed as a society-like system made by living cells, where increased survival fitness comes from large-scale cooperation among highly differentiated cells. However, cells within a multicellular mammal are mostly short-lived [1]. Therefore, a positive balance between cell death and cell proliferation is crucial for sustaining weight growth in a way similar to population curve. The weight reversal during late life represents a shift from positive to negative balance between cell proliferation and cell death, since animal weight is, in large part, determined by cell number [2]. Body composition continues to change during a protracted weight gain period (first 3 quarters of life) followed by a short weight loss period (last quarter of life) before death, representing an imbalanced development among specialized cells in an expanding and shrinking multicellular system. The relative importance of bone, muscle, and fat to sustain the longest survival duration of multicellular systems has not been strictly described by a whole-life approach observation covering the weight loss periods of end-life.

Complying with physical law, size expansion during growth will eventually reach a critical point where structural stability can no longer persist, reflected by an increase in baseline inflammation as a sign of increased entropy [3]. In humans, rapid weight gain during early adulthood is generally regarded as an adverse metabolic condition [4]. However, unintentional weight loss, that occurs during late life, is also a strong predictor of precipitous health deterioration and increased mortality [5], associated with sarcopenia, osteopenia, and lipopenia. Bone and muscle mass loss may be inter-related [6]. Bone marrow produces stem cells and immune cells which are required for tissue renewal and muscle mass gain [7]. Macrophages are bone marrow derived immune cells residing in nearly all tissues [8]. Phagocytic macrophages (CD68^+^) function to recognize and clear unhealthy senescent cells, followed by cell regeneration in presence of regenerative macrophages (CD163^+^) in muscle and all other tissues [9–11]. This system of clearance and regeneration is essential to maintain a relatively younger cell population for bone and its surrounding tissues within a multicellular system.

Insulin is essential for bone marrow cell production and bone formation [12]. The role of insulin in the regulation of muscle inflammation during the weight loss period of late life has rarely been studied. This anabolic signal stimulates daily cell regeneration suppressing entropy by lowering baseline inflammation [13]. In this study, we first examined the relationship between body composition and insulin levels every 6 months until 24 months of age in laboratory rats. Second, we determined the role of insulin on bone mass changes and bone marrow derived immune cell infiltration in the muscle of aged rats during the weight loss period at the end-of-life.

## RESULTS

**Figure 1:**
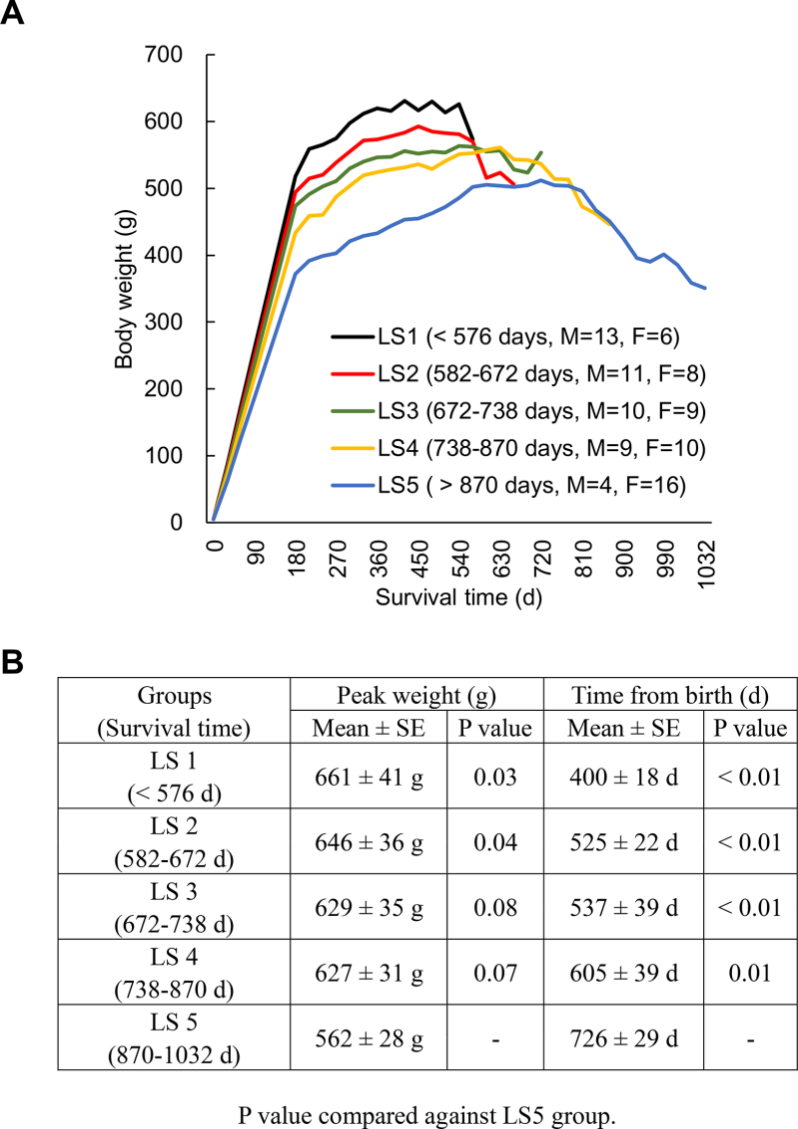
**Longevity is associated with weight trajectory patterns.** Rats (N = 96) ranked into 5 lifespan categories (LS1 to LS5), where all groups of rats show a common trajectory pattern of two discrete life phases: weight gain (long) and weight loss (short) periods (**A**). An inverse relationship between average survival time and peak weight of the groups ranked from the shortest to the longest lived group (**B**). Abbreviation: LS, lifespan; LS1 (short-lived, survival time < 579 days); LS2 (survival time 582-672 days); LS3 (survival time 672-738 days); LS4 (survival time 738-870 days); LS5 (long-lived, survival time 870-1032 days).

Figure 1A shows the weight trajectory pattern of 96 rats ranked into 5 groups according to lifespan (LS), assigned after natural death of all animals. All groups of rats show a common trajectory pattern of two discrete life phases: weight gain (long) and weight loss (short) periods. A linear (inverse) relationship between average survival time and peak weight of the groups ranked from the shortest (LS1) to the longest (LS5) lived groups was observed (Figure 1B). Long-lived rats are characterized by the modest weight gain for the first three quarters of life followed by delayed weight loss until the end of life compared to the rest groups. Female-to-male ratio of each group increases as lifespan increases. Therefore, both male and female rats were further dichotomized into fast growth group and slow growth group. Growth rates (1^st^ year) for males are 2.13 g/d (fast growth halves) and 1.83 g/d (slow growth halves) and for females are 1.12 g/d (fast growth halves) and 0.92 g/d (slow growth halves). Median lifespans and for males are 654 d (fast growth halves) and 714 d (slow growth halves) and for females are 762 d (fast growth halves) and 780 d (slow growth halves).

**Figure 2:**
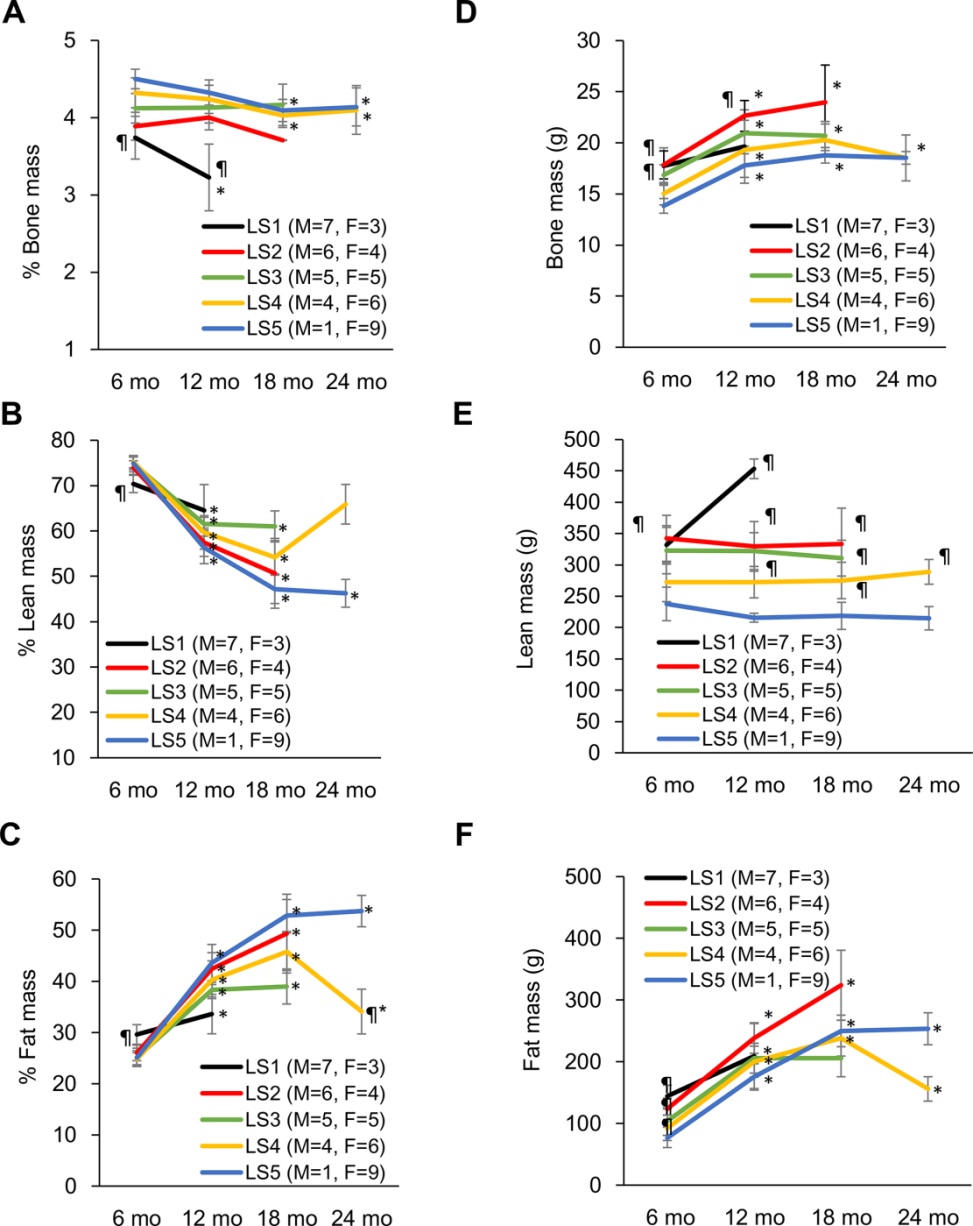
**Development of disparity in muscle, bone, and fat percentage of short-lived and long-lived rats during aging.** Long-lived rats (LS4 and LS5) are characterized by relatively higher % bone mass from 6 to 24 months of age (**A**). Short-lived rats had lowest % muscle mass (**B**) and highest % fat mass (**C**) at 6 months of age, whereas long-lived survivors showed progressive declines in % muscle mass and increases in % fat mass with age. Absolute bone mass (**D**), lean mass (**E**), and fat mass (**F**) are shown on the right side. Abbreviation: LS, lifespan; LS1 (short-lived, survival time < 579 days); LS2 (survival time 582-672 days); LS3 (survival time 672-738 days); LS4 (survival time 738-870 days); LS5 (long-lived, survival time 870-1032 days). ¶ Significant difference against long-lived rats (LS5), P < 0.05; * Significant difference against 6^th^ month, P < 0.05.

Evolutions of bone mass percentage, lean mass percentage, and fat mass percentage for the 5 groups ranked by lifespan are shown in Figure 2. At 6 months of age, the long-lived group is characterized by higher bone mass percentage (Figure 2A), higher lean mass percentage (Figure 2B), and lower fat mass percentage (Figure 2C) relative to their short-lived counterparts. However, survivors after 12 months of age show progressive decreases in lean mass percentage and bone mass percentage concurrent with increase in fat mass percentage. The most striking feature for the long-lived rats is the persistently highest bone mass percentage across the entire observation period with modest decline during end-of-life (Figure 2A). Increased lean mass percentage in the LS2 group is associated with faster fat mass loss. The absolute bone mass in the short-lived groups was relatively greater than those in the long-lived groups (Figure 2D). The lower bone mass percentage in the short-lived groups was associated with relatively greater absolute lean mass (Figure 2E) and fat mass (Figure 2F).

**Figure 3:**
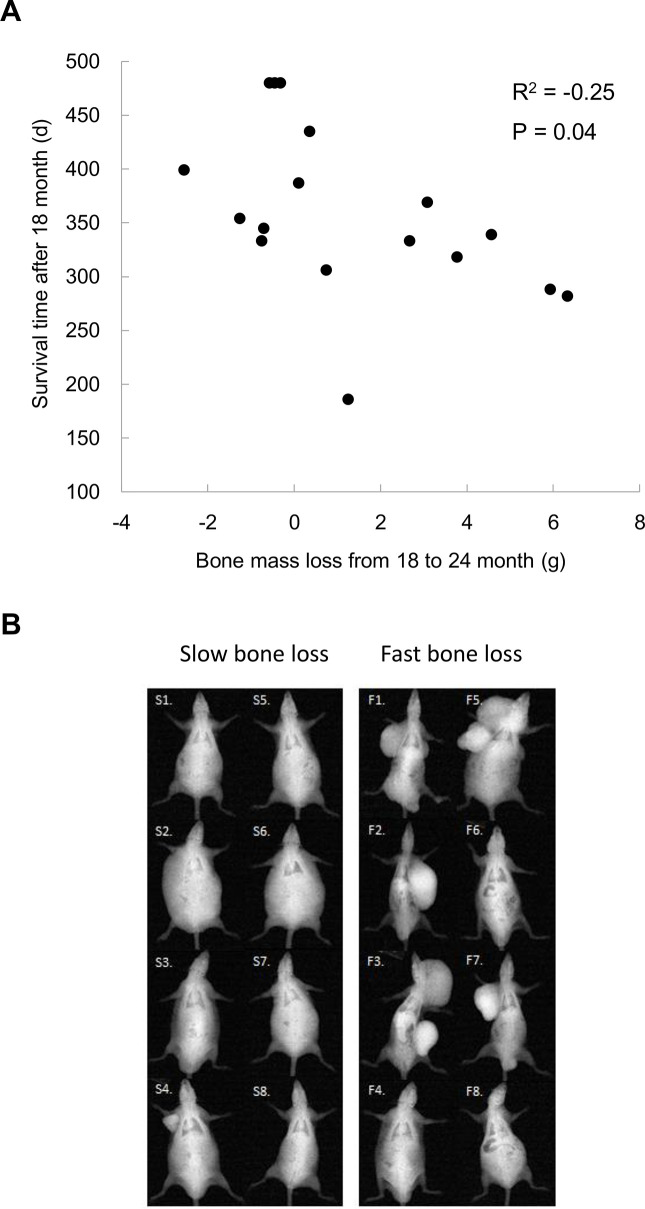
Bone mass loss is associated with tumor occurrence and residual survival time after 18 months of age (**A**). Lower panel shows x-ray images of the top 8 and bottom 8 rats (N = 17) on magnitudes of bone mass loss among rats survived after 18 months (**B**), demonstrating a severe increased tumorigenesis together with cachexia.

For those long-lived survivors (LS4 and LS5 groups), bone mass loss occurred after 18 months of age. The residual survival time after 18 months of age significantly correlated with bone mass loss including one outlier (Figure 3A). Based on X-ray image, the topmost 8 rats with the fastest bone mass loss exhibit apparently greater incidence of tumorigenesis and cachexia compared with bottommost 8 counterparts showing the slower bone mass loss during the same observation period (Figure 3B).

**Figure 4:**
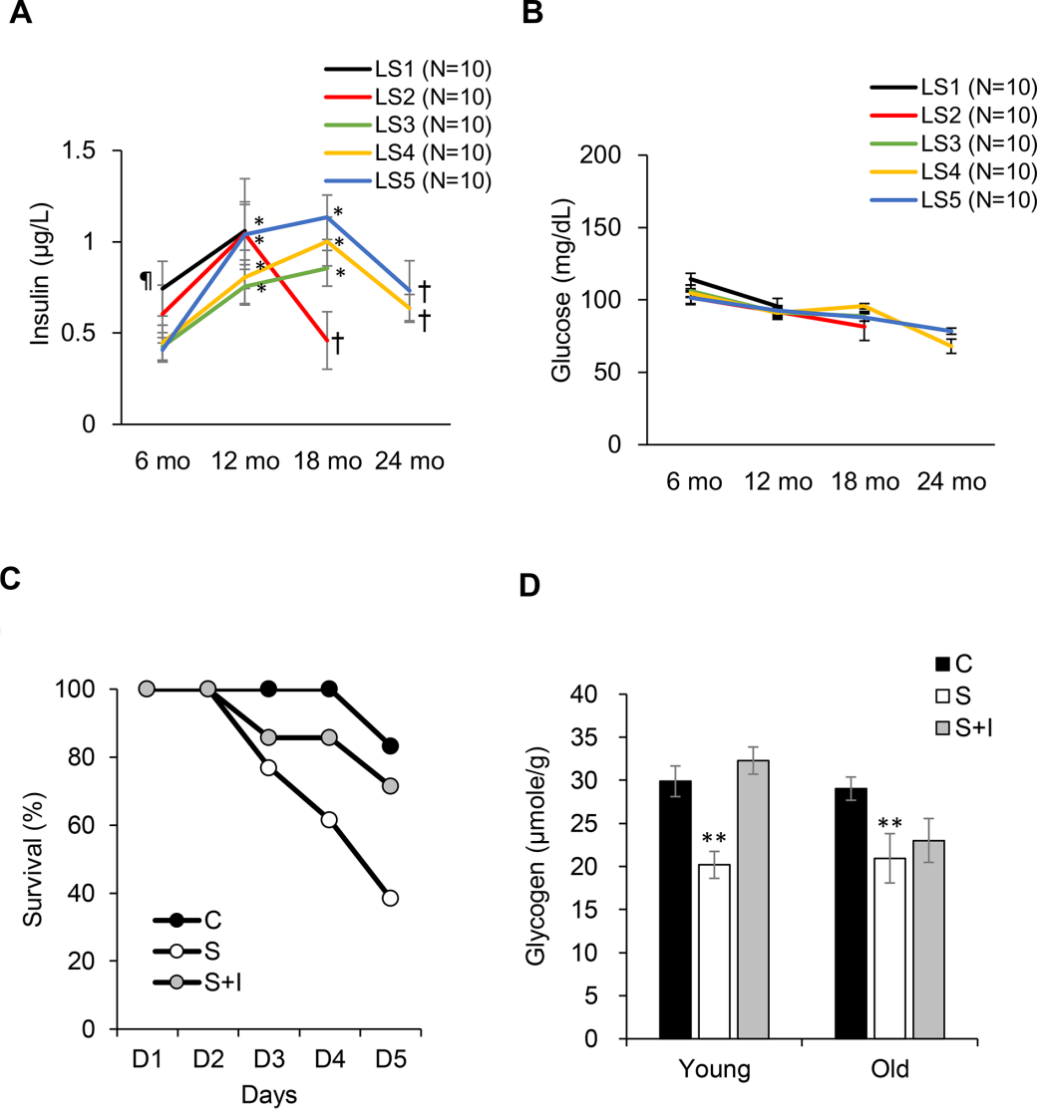
**Role of insulin on survival fitness during end-life.** Plasma insulin declines (**A**) from hyperinsulinemic state while glucose (**B**) remains unchanged from 18-24 months of age. Decreases in survival rate (**C**) and glycogen storage (**D**) after insulin loss by streptozotocin injection (S group) is reversed by insulin replacement (SI group, 0.25 IU/kg twice a day) for old rats (20 months of age). Abbreviation: LS, lifespan; LS1 (short-lived, survival time < 579 days); LS2 (survival time 582-672 days); LS3 (survival time 672-738 days); LS4 (survival time 738-870 days); LS5 (long-lived, survival time 870-1032 days). ¶ Significant difference against long-lived rats (LS5), P < 0.05; * Significant difference against 6^th^ month, P < 0.05; † Significant difference against 18^th^ month, P < 0.01. ** Significant difference against control group, P < 0.05. Abbreviation: C, vehicle-injected control group; S, Streptozotocin-injected group; SI, Streptozotocin and insulin co-injected group.

Fasting insulin levels from 6 to 24 months of age are shown in Figure 4. Short-lived rats (LS1) exhibit greatest hyperinsulinemia at 6 months of age compared with the rest groups (Figure 4A). The long-lived rats (LS4 and LS5) show delayed hyperinsulinemia and reached to similar insulin levels as the short-lived rats (LS 1 and LS2) at 18 months of age, followed by ~35% decline of insulin at 24 months of age. Glucose was not significantly different among the 5 groups and was not much changed during the 24-month period (Figure 4B). To determine the role of late-life insulin decline in lean mass loss (sarcopenia and osteopenia), STZ was injected to both young (aged 4 months) and old (aged 20 months) rats, with or without insulin co-injection. The survival rates in 5 days were 100%, 35%, and 71% for the control, STZ, and STZ + Insulin groups, respectively (Figure 4C). Survival rate of young rats (4 months of age) was not affected by STZ injection (100% survival rate). STZ eliminated plasma insulin level to an undetectable level and decreased glycogen concentration of soleus muscle by ~30%. Insulin injection to STZ-treated rats replenished muscle glycogen in young rats but had minimal effect in old rats (Figure 4D).

To determine whether insulin loss during late life is causally related with bone mass loss, the whole-body DXA scan was conducted for old rats at 20 months of age in contrast to young rats at 4 months of age after STZ injection and/or insulin replenishment. Table 1 details the changes for bone mass, lean mass, and fat mass. Body weight changes were at a rate of +1.5 ± 0.7 g per day for young rats and -7.8 ± 0.7 g per day for old rats measured 10 days before intervention. Insulin loss by STZ caused greater weight loss for old rats, while the dose of insulin injection (0.25 IU/kg twice a day) did not completely restore body weight to control level. The age-dependent weight decline occurred in parallel with lean, fat, and bone mass loss. However, bone mineral density (BMD) was not affected by the short-term insulin loss.

**Table 1:** Role of insulin on bone mass, lean mass, and fat mass in young and old rats.

**A**					
**Age**	**4-month**	**Weight (g)**	**Lean (g)**	**Fat (g)**	**Bone (g)**
Control (N = 10)	Pre	534 ±19	370±16	147±11	16.6±0.5
	Post	538±15	380±12	141±11	17.1±0.4
	∆ %	+1.2 %	+3.8 %	-3.9 %	+4.8 %*
	*Cohan’s d*	*0.07*	*0.21*	*0.15*	*0.14*
STZ (N = 10)	Pre	541±14	379±11	145±8	16.6±0.3
	Post	485±17	351±11	117±7	16.5±0.3
	∆ %	-10.5 %*	-7.5 %*	-18.7 %*	-0.5 %
	*Cohan’s d*	*1.08*	*0.78*	*1.13*	*0.10*
STZ + Insulin (N = 10)	Pre	534±11	375±11	143±7	16.4±0.3
	Post	496±11	349±9	129±6	16.9±0.4
	∆ %	-7.1 %*†	-6.6 %*	-8.9 %*†	+2.9 %*
	*Cohan’s d*	*1.15*	*0.82*	*0.65*	*0.45*

**B**					
**Age**	**20-month**	**Weight (g)**	**Lean (g)**	**Fat (g)**	**Bone (g)**
Control (N = 6)	Pre	847±18	434±10	384±9	29.3±0.9
	Post	790±11	412±6	350±10	28.7±1.2
	∆ %	-6.8 %*	-5.2 %	-8.9 %*	-2.0 %
	*Cohan’s d*	*1.16*	*0.81*	*1.08*	*0.18*
STZ (N = 5)	Pre	780±41	386±23	366±56	28.9±2.0
	Post	645±38	315±15	304±44	26.4±1.4
	∆ %	-17.3 %*	-18.4 %*	-16.8 %*	-10.5 %*
	*Cohan’s d*	*1.04*	*1.10*	*0.37*	*0.39*
STZ + Insulin (N = 6)	Pre	912±83	446±40	435±43	30.2±1.6
	Post	801±76	367±31	404±46	30.0±2.1
	∆ %	-12.2 %*†	-17.7 %*	-7.2 %*†	-0.7 %†
	*Cohan’s d*	*0.21*	*0.67*	*0.21*	*0.03*

Significant declines of bone, lean and fat occurred in 5 days after streptozotocin (STZ)-injection for rats aged at 4 months (**A**) and 20 months (**B**) of age. Following STZ-injection, all young rats survived but old rats showed high mortality rate in 5 days with a rapid weight loss. Insulin injection (0.25 IU/kg twice a day) restored bone and fat mass but not muscle mass for old rats (**B**). Non-survivors were precluded from calculation (survivor number indicated on the Table). *Significant difference against Pre, P < 0.05; † significant difference against STZ group, P < 0.05.

**Figure 5:**
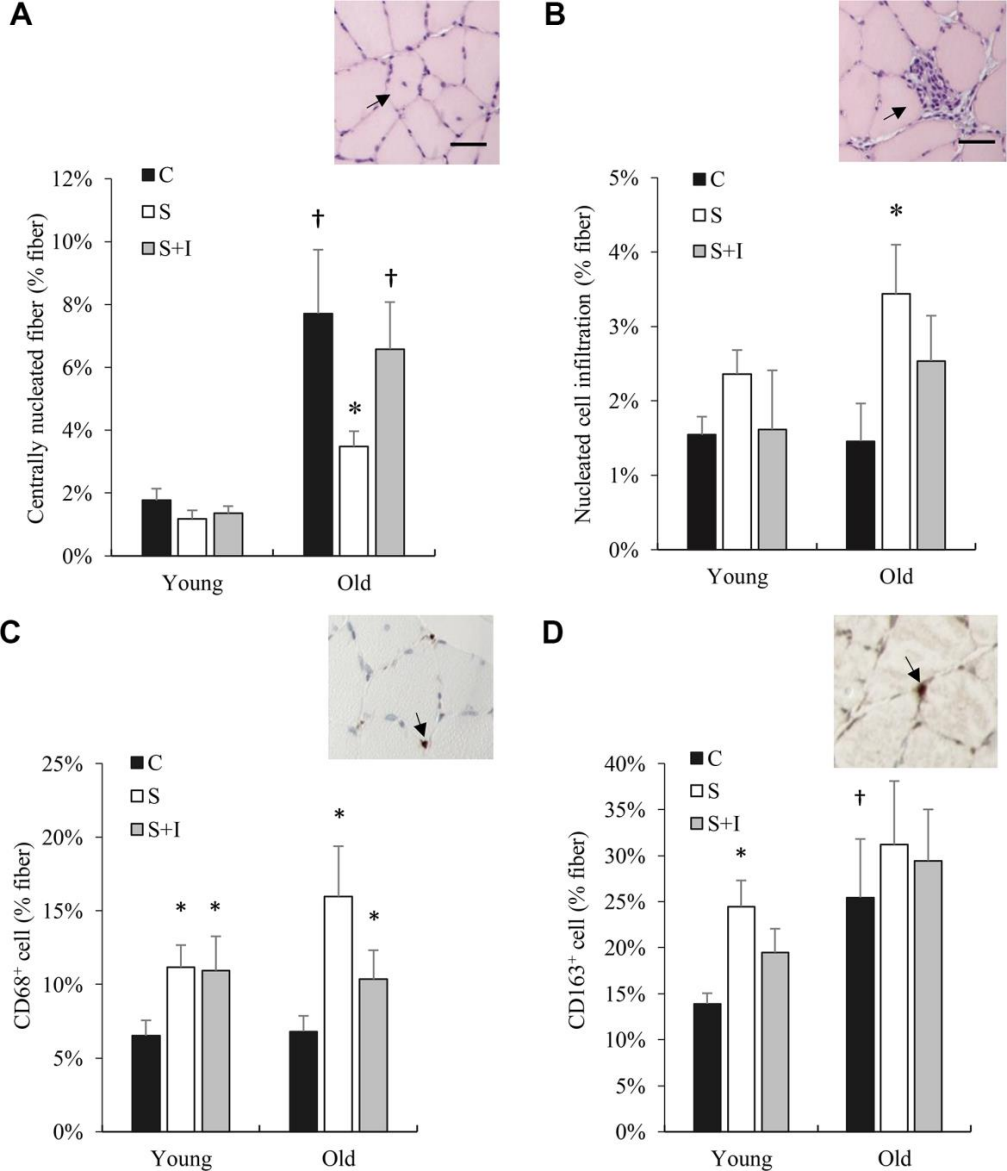
**Bone marrow derived cell infiltration (CD68^+^ and CD163^+^ cells) in skeletal muscle of old rats altered by insulin.** Centrally nucleated fibers (reflecting cell regeneration) (**A**), cell infiltration (**B**), CD68^+^ cells (**C**), and CD163^+^ cells (**D**) in soleus muscle from young and old rats were measured 5 days after streptozotocin injection (50 mg/kg/ml) and/or insulin injection (0.25 IU/kg twice a day). * Significant difference against C group, P < 0.05; † Significant against Young group, P < 0.05. Abbreviation: C, vehicle-injected control group; S, Streptozotocin-injected group; SI, Streptozotocin and insulin co-injected group.

In normal untreated rats, myofiber regeneration (centronucleation) of the 20-month rats was approximately 8-fold (Figure 5A) greater than that of the 4-month rats. Insulin depletion significantly lowered myofiber regeneration concurrent with ~50% greater cell infiltration for both young and old rats (main effect, P < 0.01) (Figure 5B). Insulin injection partially reversed these changes. Phagocytic M1 macrophage (CD68^+^ positive cells) in soleus muscle of both young and old rats was significantly increased in the STZ injected group above their control groups (P < 0.05) (Figure 5C). Regenerative M2 macrophage (CD163^+^ positive cells) in soleus muscle of old rats was substantially greater than those of young rats (Figure 5D). Insulin injection showed a small effect in reversing this change.

## DISCUSSION

Growth is an essential feature of life. However, size expansion inevitably builds up thermodynamic pressure leading to physical instability and eventual collapse of the system when the entropy of the growing body can no longer be sufficiently suppressed. In support of this notion, we observed a linear relationship between the rise-and-fall pattern of weight and lifespan among 5 groups ranked by survival time, in which the long-lived rats are characterized by the pattern of modest weight gain-and-loss trajectory during the entire life compared with the short-lived groups. This result suggests that the law of thermodynamics sets the upper ceiling of weight accumulation and thus determines the length of survival in a cell-expanding multicellular system. This seems to explain the fact that females had longer lifespan than males due to slower body size expansion. The results of the study lead to a new question whether clamping body size at youth level (i.e., at 6 months of age) preventing both weight gain and weight loss can sustain life for longer periods.

To determine the relative importance of bone, muscle, and fat on survival time of animals, we measured body composition of rats every 6 months until the end-of-life. The most striking finding of the study is the obvious linearity between bone mass percentage and the longevity of rats. Unlike other body composition variables (fat and lean mass percentage), this relationship is consistent across the weight gain and weight loss periods of life during aging. Furthermore, we observed ~ 35% decreases in plasma insulin from the hyperinsulinemic state during bone mass loss period after rats reached their peak weight in late life. We also demonstrated that insulin return from the lifetime peak levels contributes to age-dependent bone mass loss and cachexia-like condition, supported by the results of the intervention study in old rats. Insulin is essential for bone formation [14] mediated by stimulating bone marrow cell proliferation during and after daily meals [15]. Bone marrow produces immune cells (i.e. neutrophils and macrophages) for clearing unhealthy senescent cells and releases stem cells for tissue regeneration in bone and surrounding tissues [16]. Therefore, bone mass reflects the bone marrow cell producing activity, which is essential to maintain youth and size of all surrounding tissues if the balance between phagocytosis and cell regeneration can be achieved.

According to the linearity between bone mass percentage and longevity as well as the decreases in bone mass percentage after middle age, we speculate that the development of disproportionality between bone mass and body mass during growth is a likely origin of tissue aging and inevitable death during late life in multicellular mammals. Despite concurrent increases in absolute bone mass and body weight during the first 3 quarters of life, the age-dependent decreases in bone mass percentage reflect a relative deficit of the bone marrow cell producing capacity to support the increasingly larger cell population of the body. This deficit is expected to increase tissue aging due to inadequate senescent cell clearance and cell renewal.

Increasing cell populations in an expanding multicellular system will eventually reach a time point where cell death in the body rises dramatically [17], leading to an increase in baseline inflammation [3]. The result of increased insulin levels (hyperinsulinemia) during the weight gain period of early life appears to be essential to suppress inflammation in the expanding cell population of multicellular mammals [13]. Insulin is a pleiotropic anabolic hormone, which stimulates biosynthesis of DNA, glycogen, triglyceride, and protein of cells against daily catabolic challenge. At a young age, other anabolic hormones (e.g., growth hormone and sex hormones) may remain sufficiently high to suppress the thermodynamic pressure during the early weight gain period of life. For animals with increasing body weight and decreasing anabolic hormones in late life, the insulin loss is detrimental to body size maintenance and survival compared to early life.

Another main finding of the study is the higher incidence of tumorigenesis that occurred in rats with faster bone mass loss during late life. Tumorigenesis is the result of random genetic mutation during DNA replication, emerging from a high rate of cell turnover in tissues during persistent inflammation [18]. In normal tissues, cell senescence and death trigger inflammation by increasing phagocytosis and cell regeneration to ensure stable cell number and a relatively younger cell population of the tissues [19]. Tumorigenesis may reflect an imbalance between limited bone marrow cells for tissue renewal and an expanding cell population of the body. Tumors are rarely observed before middle-age. We speculate that evolution of disproportionality between bone (bone marrow cell production) and body size during late age results in senescent cell accumulation and inadequate tissue healing against daily challenges. As such, elevated senescence-induced inflammation increases cell turnover and the incidence of tumor formation.

Weight gain during the early period of life together with hyperinsulinemia has been recognized as a common predictor of age-related metabolic conditions and premature death [20, 21]. In this study, we have further found insulin reversal from its peak in absence of glucose elevation during the weight loss period at the end-of-life. One possible explanation is that there is an increased basal glucose uptake due to a higher rate of cell regeneration. This is revealed by increased centrally nucleated fibers in aged muscle. Newly generated muscle progenitor cells exhibit much greater rate of basal glucose transport (mediated by GLUT1 protein) than mature myofibers [22]. However, insulin stimulated glycogen storage (mediated by GLUT4) was blunted. Our finding demonstrating an increased myofiber regeneration in aged muscle is consistent with a previous study [23]. Insulin loss during this period can exacerbate exhaustion of bone marrow cells by unhealthy muscle tissues which results in bone mass loss and sarcopenia.

The major limitation of the study is that DXA could not distinguish muscle from fibrotic tissue or tumor in a whole-body scan for aged animals [24]. This technical limitation may explain the absence of lean mass recovery after insulin injection in the STZ treated rats despite a rapid recovery of fat and bone mass. For the future study, development of an accurate appendicular muscle assessment technique for animal imaging systems may be helpful to resolve the technical limitations of DXA. Furthermore, the results of the insulin intervention study in reversing bone mass loss and preserving survival of STZ-treated old animals should not be generalized to encourage insulin supplementation for middle-aged individuals during weight gain period of life.

## CONCLUSIONS

Bone produces stem cells and phagocytes which are able to maintain the youth of cells (prevent the senescence) for bone itself and neighboring tissues. Development of disproportionality between bone mass and body mass during growth is closely associated with aging and unsustainable life. Aging skeletal muscle exhausts bone marrow cells particularly when insulin returns from its lifetime peak, which contributes osteopenia and the eventual death of old rats.

## MATERIALS AND METHODS

### Ethical statement

All experimental procedure followed Animal Protection Act of Taiwan and approved by the Institutional Animal Care and Use Committee of the University of Taipei, Taipei, Taiwan.

### Animal care

Sprague–Dawley rats were obtained from BioLASCO Co., Ltd, Taipei City, Taiwan. They were housed in animal facility at University of Taipei. All animals were maintained in a temperature-controlled room (21 ± 2° C) under a 12-h light–dark cycle (6:00 a.m.–6:00 p.m.) and a relative humidity of 45-55%. Every 2 animals were housed for each cage. They were fed with standard rat chow (Rodent Diet 5001, LabDiet, St. Louis, MO, USA) and normal tap water in feeding bottle, recorded the amount of food intake and body weight at the same time twice a day. Food and water intake were recorded.

### Longevity study

This study aimed to characterize body composition trajectories of long-lived and short-lived rats. One hundred Sprague-Dawley rats at one month of age with known date of birth were transferred to animal facility at University of Taipei in 2012 (N =50) and 2016 (N = 50) for entire life until natural death. Animals were ranked into 5 groups and subsequent data were created following the death of the animals used in the study. The long-lived and the short-lived groups were treated equally aside from the obvious continued treatment and the data of these long- and short-lived animals were compared retrospectively. The animals included in insulin trajectory age-matched to the initial study were treated equally and assigned to long- or short-lived groups post-mortem. Linearity between bone mass percentage (bone mass divided by body mass in percentage) and longevity (survival time) among 5 ranked group was first established in the first experiment in 2012. The second study in 2016 used the same number of rats to determine reproducibility of the first observation, which showed similar pattern. Taken together, a total of 96 rats (M = 47; F = 49) were included to examine lifetime body weight changes among 5 ranked group according to their lifespan. The second half of animals was used to further determine insulin trajectories for the age-matched comparison among the 5 groups. To study aging associated death, rats survived < 12 months (N = 4) were excluded. For the repeated experiment, a total of 50 rats (M = 23; F = 27) was used for insulin analysis. After a 12-h fasting, blood sample (0.15 mL) were collected at 6, 12, 18, and 24 months of age for glucose and serum insulin measurements to obtain trajectory. The fluid loss is compensated by the same volume of intraperitoneal injection (0.15 mL) of sodium pentobarbital (80 mg/kg) to minimize the potential influence on DXA assessment. Body composition was measured following blood collection. There were 26 rats survived until the last measurement at 24 months of age.

### Intervention study

An intervention study was designed to examine the causal effect of insulin change on body composition (% lean, fat, and bone mass) and muscle inflammation during weight loss period of late life. Weight loss rate was measured 10 days before treatments. Since weight loss is highly associated with increased mortality among aged animals, 30 rats were weight-matched into 3 groups. Additional 6 rats were allocated to the S group as STZ is expected to produce high mortality rate to aged rats. Control group (C, N = 8) received intraperitoneal injections of 0.1 M citrate buffer solution. Two rats in the C group died before intervention after grouping were precluded from mortality analysis. STZ group (S, N = 14) received intraperitoneal injections of STZ (50 mg/kg/ml in 0.1 M citrate buffer solution); STZ with insulin group (SI, N = 7) received intraperitoneal insulin injection (0.25 IU/kg in 0.1 M citrate buffer solution) every 12 h right after STZ injection. Due to high mortality among the aged rats, only the animals with complete data after tissue collection were included (C: N = 6; S, N = 5; SI: N = 6) for tissue analysis and pre-post comparison of body composition measures. Same number of young rats (N = 30) at 4 months of age was used as age control and was evenly assigned to each group (N = 10). All rats were survived after treatments. Both insulin and vehicle solution were delivered daily at the same time of the day for treated rats. Soleus muscle was used for glycogen analysis and immunohistochemical analysis on myeloid cell infiltration.

### Streptozotocin and insulin

Streptozotocin (Sigma-Aldrich, Darmstadt, Germany) was dissolved in 0.1 M citrate buffer at the dose of 50 mg/kg body weight to eliminate insulin to undetectable level. Insulin (Humulin®, Lilly, USA) was diluted with saline and injected 0.25 IU/kg every 12 h. Animals were fasted overnight for 12 h before STZ injection. Food and water were made available immediately after injection. Loss of insulin was confirmed by measurements of serum insulin using enzyme-linked immunosorbent assay (Mercodia, Uppsala, Sweden) 5 days after STZ injection. Responsiveness of rats (SI group) to human insulin was confirmed by muscle glycogen concentration 3 h after glucose intubation with insulin injection. Survived rats were anesthetized with sodium pentobarbital (80 mg/kg) 5 d after intervention. Soleus muscle was surgically collected for immunohistochemically analysis and glycogen assay.

### Body composition

Body composition was conducted under overnight fasted condition. Dual-energy x-ray absorptiometry (DXA) (Lunar iDXA, GE Medical Systems, Madison, WI) with resolution = 1 (line-pair/mm) and pixel size = 0.5 mm was used for whole-body scans every 6 months for both the longevity study and the intervention study. Precision errors of the DXA are BMD 1.08%, BMC 1.00%, fat mass 1.22%, lean mass 0.95%, and percent fat 0.78%. To ensure DXA functionality, phantom calibration and quality assurance procedure were performed before scans. Before taken dual-energy x-ray absorptiometry, each rat was given an intraperitoneal injection of sodium pentobarbital (80 mg/kg) and position fixed on the platform by taping. Food and water were removed 12 h before measurement.

### Glycogen assay

To determine insulin responsiveness to human insulin injection, muscle glycogen was measured in soleus muscle. Soleus muscle was frozen in liquid nitrogen until glycogen analysis. Muscle glycogen content was broken down into glucose units using amyloglucosidase (Teco Diagnostics, Anaheim, CA, USA) to determine the amount of glycogen as glucose unit (55 mg of averaged muscle weight). The experimental protocol followed our previous study (Kuo, Browning, and Ivy, 1999). Glucose concentration was measured by reading the optical density at 505 nm by spectrophotometer (Thermo, Genesis 10S UV-Vis, NY, USA) after 18 minutes.

### Immunohistochemical (IHC) analysis

Histology for leukocyte infiltration and IHC staining for macrophage infiltration were conducted by a professional pathologist at Taipei Institute of Pathology (Taipei, Taiwan). Middle section of soleus muscle was placed in 10% solution of formaldehyde for no more than 3 h before analysis. Soleus muscle was fixed in 4% formalin and then embedded in paraffin. All soleus sample in paraffin were sliced in serial cross-sections (3 μm each) for hematoxylin-eosin staining and immunohistochemistry analyses. For each sample, more than 500 myofibers were included for analysis. Tissue sections were transferred onto coated slides (Super Frost Plus, Braunschweig, Germany). Antigen retrieval occurred in boiled water for 15 min in 0.1 M sodium citrate (pH 7.2). These pretreated slides were blocked for 15 min at room temperature with 5% BSA and then incubated at 4° C overnight with primary antibodies. Antibodies against rat CD68 (dilution 1:50) (abcam, Cambridge, UK) and rat CD163^+^ (dilution 1:100) (Bio-Rad, Hercules, USA). Briefly, specific antibody was purchased to perform IHC staining by using horseradish peroxidase-conjugated avidin biotin complex (ABC) from the Vectastain Elite ABC Kit (Vector Laboratories, Burlingame, CA, USA) and DAB chromogen (Vector Laboratories). The sections were counterstained with hematoxylin and mounted. Macrophage infiltration are expressed as positive cell number per myofiber.

### Statistical analysis

Analysis of variance (ANOVA) was used to compare difference among groups for all variables. A one-way analysis of variance (ANOVA) with a repeated measure was used to compare the mean differences in body composition variables between pre- and post-treatments. Fisher’s protected least significant test, which holds the value of type I error to 5% for each test, was used to distinguish the differences between pairs of groups. All values are expressed as means ± standard errors. All statistical analyses were performed using SPSS.

### Data availability statement

This data will be made available from authors upon reasonable request.

## References

[1] Spalding KL, Bhardwaj RD, Buchholz BA, Druid H, Frisén J. Retrospective birth dating of cells in humans. Cell. 2005; 122:133–43. 10.1016/j.cell.2005.04.028 16009139

[2] Conlon I, Raff M. Size control in animal development. Cell. 1999; 96:235–44. 10.1016/s0092-8674(00)80563-2 9988218

[3] Fogarty AW, Glancy C, Jones S, Lewis SA, McKeever TM, Britton JR. A prospective study of weight change and systemic inflammation over 9 y. Am J Clin Nutr. 2008; 87:30–35. 10.1093/ajcn/87.1.30 18175734

[4] Sun K, Kusminski CM, Scherer PE. Adipose tissue remodeling and obesity. J Clin Invest. 2011; 121:2094–101. 10.1172/JCI45887 21633177 PMC3104761

[5] Alley DE, Metter EJ, Griswold ME, Harris TB, Simonsick EM, Longo DL, Ferrucci L. Changes in weight at the end of life: characterizing weight loss by time to death in a cohort study of older men. Am J Epidemiol. 2010; 172:558–65. 10.1093/aje/kwq168 20682520 PMC3025636

[6] Ferrucci L, Baroni M, Ranchelli A, Lauretani F, Maggio M, Mecocci P, Ruggiero C. Interaction between bone and muscle in older persons with mobility limitations. Curr Pharm Des. 2014; 20:3178–97. 10.2174/13816128113196660690 24050165 PMC4586132

[7] Tidball JG. Regulation of muscle growth and regeneration by the immune system. Nat Rev Immunol. 2017; 17:165–78. 10.1038/nri.2016.150 28163303 PMC5452982

[8] Schindeler A, McDonald MM, Bokko P, Little DG. Bone remodeling during fracture repair: the cellular picture. Semin Cell Dev Biol. 2008; 19:459–66. 10.1016/j.semcdb.2008.07.004 18692584

[9] Chang MK, Raggatt LJ, Alexander KA, Kuliwaba JS, Fazzalari NL, Schroder K, Maylin ER, Ripoll VM, Hume DA, Pettit AR. Osteal tissue macrophages are intercalated throughout human and mouse bone lining tissues and regulate osteoblast function *in vitro* and *in vivo*. J Immunol. 2008; 181:1232–44. 10.4049/jimmunol.181.2.1232 18606677

[10] Gu Q, Yang H, Shi Q. Macrophages and bone inflammation. J Orthop Translat. 2017; 10:86–93. 10.1016/j.jot.2017.05.002 29662760 PMC5822954

[11] Tidball JG. Inflammatory processes in muscle injury and repair. Am J Physiol Regul Integr Comp Physiol. 2005; 288:R345–53. 10.1152/ajpregu.00454.2004 15637171

[12] Weinberg E, Maymon T, Moses O, Weinreb M. Streptozotocin-induced diabetes in rats diminishes the size of the osteoprogenitor pool in bone marrow. Diabetes Res Clin Pract. 2014; 103:35–41. 10.1016/j.diabres.2013.11.015 24314392

[13] Dandona P, Chaudhuri A, Ghanim H, Mohanty P. Insulin as an anti-inflammatory and antiatherogenic modulator. J Am Coll Cardiol. 2009; 53:S14–20. 10.1016/j.jacc.2008.10.038 19179212

[14] Sinder BP, Pettit AR, McCauley LK. Macrophages: their emerging roles in bone. J Bone Miner Res. 2015; 30:2140–49. 10.1002/jbmr.2735 26531055 PMC4876707

[15] Miyagawa S, Kobayashi M, Konishi N, Sato T, Ueda K. Insulin and insulin-like growth factor I support the proliferation of erythroid progenitor cells in bone marrow through the sharing of receptors. Br J Haematol. 2000; 109:555–62. 10.1046/j.1365-2141.2000.02047.x 10886204

[16] Kratofil RM, Kubes P, Deniset JF. Monocyte conversion during inflammation and injury. Arterioscler Thromb Vasc Biol. 2017; 37:35–42. 10.1161/ATVBAHA.116.308198 27765768

[17] Ratcliff WC, Denison RF, Borrello M, Travisano M. Experimental evolution of multicellularity. Proc Natl Acad Sci USA. 2012; 109:1595–600. 10.1073/pnas.1115323109 22307617 PMC3277146

[18] Dvorak HF. Tumors: wounds that do not heal. Similarities between tumor stroma generation and wound healing. N Engl J Med. 1986; 315:1650–9. 10.1056/NEJM198612253152606 3537791

[19] Lasry A, Ben-Neriah Y. Senescence-associated inflammatory responses: aging and cancer perspectives. Trends Immunol. 2015; 36:217–28. 10.1016/j.it.2015.02.009 25801910

[20] Ausk KJ, Boyko EJ, Ioannou GN. Insulin resistance predicts mortality in nondiabetic individuals in the U.S. Diabetes Care. 2010; 33:1179–85. 10.2337/dc09-2110 20200308 PMC2875420

[21] Facchini FS, Hua N, Abbasi F, Reaven GM. Insulin resistance as a predictor of age-related diseases. J Clin Endocrinol Metab. 2001; 86:3574–78. 10.1210/jcem.86.8.7763 11502781

[22] Mitsumoto Y, Burdett E, Grant A, Klip A. Differential expression of the GLUT1 and GLUT4 glucose transporters during differentiation of L6 muscle cells. Biochem Biophys Res Commun. 1991; 175:652–59. 10.1016/0006-291x(91)91615-j 2018509

[23] Wang Y, Wehling-Henricks M, Samengo G, Tidball JG. Increases of M2a macrophages and fibrosis in aging muscle are influenced by bone marrow aging and negatively regulated by muscle-derived nitric oxide. Aging Cell. 2015; 14:678–88. 10.1111/acel.12350 26009878 PMC4531081

[24] Szulc P, Munoz F, Marchand F, Chapurlat R, Delmas PD. Rapid loss of appendicular skeletal muscle mass is associated with higher all-cause mortality in older men: the prospective MINOS study. Am J Clin Nutr. 2010; 91:1227–36. 10.3945/ajcn.2009.28256 20237137

